# Does maturity estimation, 2D:4D and training load measures explain physical fitness changes of youth football players?

**DOI:** 10.1186/s12887-022-03801-5

**Published:** 2022-12-20

**Authors:** Rui Miguel Silva, Filipe Manuel Clemente, Francisco González-Fernández, Hadi Nobari, Hamed Haghighi, José Ma. Cancela Carral

**Affiliations:** 1 Faculty of Educational Sciences and Sports Sciences, University of Vigo, 36005 Pontevedra, Spain; 2 Escola Superior Desporto e Lazer, Instituto Politécnico de Viana do Castelo, Rua Escola Industrial e Comercial de Nun’Álvares, 4900-347 Viana do Castelo, Portugal; 3 Research Center in Sports Performance, Recreation, Innovation and Technology – SPRINT, 4900-347 Viana do Castelo, Portugal; 4 Department of Physical Education and Sport, Faculty of Education and Sport Sciences, University of Granada, Campus Melilla, 52006 Melilla, Spain; 5 SER Research Group, Pontifical University of Comillas, 07013 Palma, Spain; 6 Faculty of Sport Sciences, University of Extremadura, 10003 Cáceres, Spain; 7 Department of Exercise Physiology, Faculty of Educational Sciences and Psychology, University of Mohaghegh Ardabili, 56199-11367 Ardabil, Iran; 8 Department of Motor Performance, Faculty of Physical Education and Mountain Sports, Transilvania University of Brasov, 500068 Brasov, Romania; 9 Department of Sports Injuries and Corrective Exercises, Faculty of Sport Sciences, University of Isfahan, 81746-7344 Isfahan, Iran

**Keywords:** Maturation, Assessment, Monitoring, Performance

## Abstract

**Objectives:**

The purpose of the present study was two-fold: (1) To analyse physical fitness changes of youth football players after a full-season; and (2) to examine whether physical fitness changes are explainable by estimated maturity status, 2digit:4digit ratio (2D:4D) from each hand and training load (TL) measures.

**Methods:**

Twenty-seven youth elite Under-15 football players were daily monitored for training load measures during 38 weeks. At the beginning and at the end of the season, all players were assessed for physical fitness. Also, the maturity status estimation and the length of the second and fourth digits of both hands were collected at the beginning of the season.

**Results:**

Significant differences were found for all physical fitness measures after the season. The second and fourth digits of left and right hands had negative moderate correlations with change of direction (COD) changes (*r*=-.39 to − 0.45 | *p* = .05 to 0.02). Also, the maturity offset measure had negative moderate correlations with COD changes (*r*=-.40 | *p* = .04). From the reported significant correlations, the maturity offset, Left 4D, Right 2D and Right 4D significantly predicted the Mod.505 COD test changes (β = 0.41, *p* = .04; β = -0.41, *p* = .04; β = -0.45, *p* = .02; and β = -0.44, *p* = .03, respectively).

**Conclusion:**

The maturity offset and the 2D:4D measures have the potential to predict COD performance changes over-time in youth football players. Given the lack of associations between the maturity estimation, 2D:4D and training load measures, with the overall physical fitness measures, coaches should rely only at COD changes.

## Background

The physiological and physical characteristics of academy football players are well described at different age-categories [1, 2]. For instance, youth football players may present VO_2_max values as high as approximately 67 ml/kg/min and lactate concentrations that can be between 2 and 11.9 mmol·L^-1^ during a football match [3]. During a youth football match, players spent a great proportion of playing time above 80% of their individual maximal heart rate (measured by a cardiorespiratory test until exhaustion), for all age-categories [4]. Also, the anaerobic power values (measured by the Wingate Anaerobic Test (WAnT), can reach to approximately 11 W·kg^-1^ in youth football players [5].

To cope with football match physiological and physical demands, youth players must gradually develop great physical fitness levels [6]. While a focus on the development of technical skills, agility and running speed seem to be more important in the U13 and U14 age-categories, the development of cardiorespiratory capacity can be crucial in U15 and U16 academy football players [7]. Also, at those age-categories (U15 and U16), the development and demonstration of strength, lower-limbs power and sprint performance assume imperative roles during match performance [8]. In fact, it was previously demonstrated that higher levels of lower-body strength showed strong associations with better sprint and jump performance in youth football players [8]. Furthermore, match running performance and the physical capacities described above seem to be related to each other, although its magnitude tends to be different when categorizing the players into the different on-field playing positions [9]. Moreover, field tests aerobic performances such as during the Yo-Yo Intermittent Recovery (YYIR) test, are related with the capacity to spend more time executing high-intensity activities during a football match [10].

A football season is usually divided by three different moments (e.g., pre-season, in-season, and off-season), where it is expected that both youth and adult players present significant variations in terms of physical fitness during those different periods of the season [11]. However, it seems to persist some incongruences in literature regarding physical fitness changes throughout a football season [12, 13]. Despite that, it is well described that the pre-season is considered an important period where it is supposed to occur significant physical fitness improvements [14]. Those improvements can be even more pronounced if a training plan during the off-season period was not well prescribed for players of the same team [11]. Furthermore, during the in-season period slight improvements can be observed in some players, although there is a tendency for a maintenance of physical status during the in-season, followed by decreases during the final stages of the season [15]. The training-dose imposed to youth football players, and their accumulated perceived intensity of training, can explain at some magnitude, the observed physical fitness changes after a determined period of training [16].

Youth players’ maturational status might also influence youth players physical fitness changes throughout a full-season [17]. The biological development of males during the adolescence, usually starts peaking at approximately 13/14 years of their chronological age [18]. However, this can vary significantly if it is considered the biological age of players. That is, players of the same chronological age can be at different levels of maturation [19]. Players that are more advanced in maturity status compared to their same-age colleagues, usually present greater physical performance mainly in strength manifestation as a consequence of greater increases in muscle mass [20]. On the other hand, more mature players in the U14 and U15 age-categories, can express the so-called “motor-awkwardness”, that may limit their performance in other tasks, such as technical skills [21]. Although an influence of the relative age effect is present on the birth-date distribution of players from U14 and U16 age-categories, a previous study revealed a lack of significant associations between relative age effect and the selection process [22].

Given that, it is of paramount importance to measure the maturity status of youth football players within the same team. One of the most used methods to estimate player’s maturity status in team sports context is the maturity offset [23]. The maturity offset is a non-invasive method, based on anthropometric measures that allows to estimate at what age a young player will achieve the peak height velocity (PHV) [23]. This method was previously shown to be valid and reliable, however, its inability to differentiate between early and late maturing youth is a limitation to be considered [18]. Despite that, recent research revealed that PHV estimations showed that increases in maturity status results lower perceived intensity during training in youth football players [24].

A recent study conducted on 88 youth football players from different age-categories (U12, 13, 14 and 15), revealed that the accumulated training, maturity and initial physical fitness status explained only small and inconsistent proportions of the observed physical fitness changes after a full-season [25]. Another study conducted on 68 youth football players from different age-categories revealed that player’s maturation status have a moderate effect on match work rate [26]. On the other hand, biological maturity was previously associated with global positioning system running measures only for U14 and not for U15 and U16 age-categories [27].

The length of the hand second digit divided by the length of the fourth digit is known as 2D:4D [28]. This marker of prenatal testosterone exposure has been previously shown to be a potential predictor of physical performance [29]. Indeed, a study conducted on 24 youth players found that the 2D:4D of both hands had large negative correlations with VO_2_max and strength changes, and showed to be a good predictor of the mentioned physical changes in youth male football players [30]. Another recent study that examined the relationships between 2D:4D, aerobic fitness, physical skills, and overall physical fitness male and female runners, revealed that males with lower right hand 2D:4D had greater values of VO_2_max and point of equivalent change [31]. These findings suggest that using the 2D:4D can give potential insights regarding players’ physical performance. However, it was previously shown that the 2D:4D did not reveal significant associations with changes in training load throughout a youth football season [30]. Also, sports scientists and practitioners must be aware to the fact that limitations were previously reported when using the 2D:4D for size-scaling and between-group comparisons [32].

Few studies focused on the potential of maturity estimation and of 2D:4D measures to explain physical fitness changes of youth football players after a full-season [25, 33]. Given the findings of previous research [31], it is hypothesized that lower 2D:4D would predict changes in overall physical fitness measures in young football players. For those reasons, the present study aims to analyse physical fitness changes of youth football players after a full-season; and to examine whether physical fitness changes are explainable by maturity status estimation, 2D:4D from each hand and TL measures.

### Materials and methods

#### Design and procedures

**Fig. 1:**
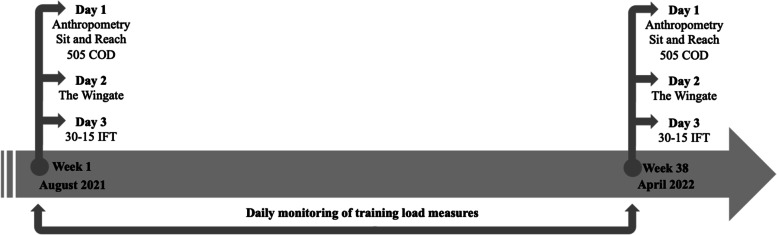
The timeline of the two moments of assessments. 505 COD: 505 change of direction test; 30-15IFT: 30 − 15 intermittent fitness test

A prospective cohort study design was used. Players from a single under-15 youth male football team were prospectively analyzed throughout 38 weeks of a competitive season. Anthropometric and body composition measures were conducted. Also, the maturity status of each player was assessed using anthropometric data. Physical assessments were carried out in August 2021, and in April 2022. The participants were assessed during three days. Anthropometry, body composition, range of motion and change of direction (COD) assessments were carried out in the first day. The assessment of anaerobic performance was made during the second day, using the Wingate test. In the third day of assessments, the 30 − 15 intermittent fitness test (30 − 15 IFT) was conducted to assess the participant’s aerobic performance. Only the COD and the 30 − 15 IFT assessments were conducted outdoors, on a synthetic turf soccer field. The timeline of the two moments of assessments are illustrated in Fig. 1.

### Participants

Twenty-seven youth male football players (age: 15.0 ± 0.4 years old; height: 175 ± 0.6 cm; body mass: 62.1 ± 7.0 kg; V02_max_: 45.0 ± 4.3 mL^·^kg ^-1·^min ^− 1^) from the same team and competing in the national under-15 championship participated in this study. The inclusion criteria were: (i) all players had to participate in at least 90% of training sessions throughout the season; (ii) for each week, the players had to participate in all training sessions; and (iii) not be injured during the observations and assessments. The goalkeepers were excluded from the sample. Before the beginning of this study, all participants and their parents or their legal representants signed a written informed consent form. Thus, all the advantages and disadvantages of the study procedures were well explained to all the involved. The present study followed the ethical recommendations for the study in humans as suggested by the Declaration of Helsinki (updated version from 2013).

### Training load quantification

Training load was collected using the rate of perceived exertion (RPE) based on the CR-10 Borg scale [34]. Thus, the RPE values were collected approximately 10–30 min after each training session, as recommended in previous research [35]. Based on the CR-10 scale, 1 means “very light activity” and 10 means “maximal exertion”. All players answered to the question “How intense was your session?”. Their responses were given in an individual way, and without the influence of their colleagues. Additionally, the duration of the training sessions, in minutes, was recorded. After obtaining each player RPE value, the session-rate of perceived exertion (s-RPE) was used [36]. To obtain the s-RPE values, the duration of each training session was recorded and multiplied by the RPE value attributed by each player, and was presented as an arbitrary unit (A.U.). Moreover, from the s-RPE values, the weekly training load (wTL, sum of the load of all sessions and match), mean training load (mTL, mean of the load of all sessions and match), 5-day average (5d-AVG, mean of the load of five training sessions without match), the training monotony (TM, mean of training load of 7 days divided by the standard deviation), and the training strain (TS, sum of the load of all sessions and match multiplied by training monotony per week) were calculated.

### Physical fitness assessments

#### Anthropometry and body composition

Standing stature was measured using a stadiometer stadiometer (Seca model 213, Germany) with an accuracy of ± 5 mm and body mass was measured using a balance (Seca model 813, UK) with a precision of 0.1 per kilogram. All players were assessed without shoes and with their lower back as close to the stadiometer as possible. For measuring body composition, three-point skinfolds (chest, abdominal and thigh) were conducted to measure the body fat percentage (BF%). All skinfold measures were assessed using a Lafayette caliper (Lafayette, IN, USA) with an accuracy of 0.1 mm. The skinfold measurements were applied twice on the right side of the athlete’s body, and the final score recorded were the mean of two measurements. If the measurement error was high (> 5%), the measurements had to be performed again and the median of the three repetitions were used for analysis. All measurements were performed by an ISAK credited person. Thus, the calculations of body density and body fat% were calculated based on the Jackson and Pollock formula [37].

### Sit and reach test

For the estimation of hamstring extensibility, the Sit and Reach test was conducted. This test was previously had a moderate criterion-related validity for estimating hamstring extensibility (*r* = .46–0.67) [38]. All participants had to sit on the floor with their bare feet against the sit-and-reach equipment and with their middle fingers stacked on top of one another. Participants were informed to stretch as far as possible without bending their knees. The final outcome to be used was the distance between the tip of the middle fingers and the toe line, as previously recommended [39].

### Modified 505 COD test

For measuring the participants ability to change directions, the modified 505 COD test was used as in elsewhere [40]. This test was previously considered valid and reliable [41, 42]. Three cones were placed at 5-meters apart from each other, and a pair of photocells with a digital timer connected to it was placed at cone B. The photocell system used was the Newtest Power timer 300-series, that was adjusted to each player’s hip height. Each participant started the test 70 cm before the cone A (starting line). After a beep sound, each participant had to run as quickly as possible until reaching cone C, turn on the cone C line and return as quickly as possible through the photocells (cone B). Test time was measured to the nearest 0.01 s with the fastest value obtained from 2 maximal trials. After each trial, the players had a 3-minute recovery.

### Wingate test

For measuring the anaerobic performance of each participant, the Wingate Anaerobic Test (WAnT) was performed on a cycle ergometer (Monark model 894-E, Vansbro, Sweden). During 5 s, the participants had to pedal at maximum speed to determine the repetition per minute (RPM) in the ergometer monitor. After that, a braking force was determined by the product of body mass in kg by 0.075. The participants had to pedal at their maximum effort during 30 s with verbal encouragement from the coach and/or colleagues. The peak power (PP) measure was used for further analysis [43].

### 30 − 15 intermittent fitness test

For measuring maximal cardiorespiratory function, anaerobic capacity, neuromuscular function and the ability to recover during intermittent exercise, the 30 − 15 Intermittent Fitness test (30 − 15 IFT) was applied. The test initial velocity was set at 8 km.h^− 1^ during the first run, and was increased by 0.5 km/h^-1^ after each running sequence. All participants had to run back and forth within a 40-meter straight line. Each shuttle consists of 30-second runs interspersed with 15-seconds of walking. 3-meter zones were delineated in both extremities and at the middle of the test setup. Each participant had to complete as many stages as possible, and the test ended when the players could not maintain the running speed demanded, or could not reach the 3-meter zone before the beep during three times. As a final outcome to be analyzed, the velocity of intermittent fitness test (VIFT) score of each participant was recorded. The VIFT consists of the final velocity recorded during the last stage. Also, the VO_2_max was estimated by the following equation for each player [44]: Estimated VO_2_max = 28.3 −(2.15 × 1) −(0.741 × age) −(0.0357 × mass) + (0.0586 × age × VIFT) + (1.03 × VIFT).

The test-retest reliability of the physical fitness measures included in this study used were tested (Table 1).

**Table 1:** Test-retest reliability of all fitness measures

Fitness measures	ICC	95%CI [lower;upper]	*p*
**Sit and Reach**	0.978	0.785;0.976	*p <* .001
**505 COD**	0.708	0.053;0.728	*p <* .001
**Peak Power**	0.873	0.300;0.962	*p <* .001
**VIFT (Km/h)**	0.948	0,761;0.983	*p* = .001

505 COD: 505 change of direction, *ICC* intraclass correlation coefficient, *CI* confidence interval

### Maturity offset and age at PHV

To estimate the age at peak heigh velocity (PHV) of each player, the maturity offset was calculated using the chronological age, standing height, sitting height, leg length and body weight measures, according to the following equation [45]: Maturity Offset = − 9.236 + 0.0002708 (leg length × sitting height) − 0.001663 (chronological age × leg length) + 0.007216 (chronological age × sitting height) + 0.02292 (mass by height ratio). For measuring the sitting height, the athletes were asked to sit on the 50 cm height box, facing forwards. Then the height between the highest point of the head and the bottom of the box the player was sitting in, was measured. For measuring the leg length, the standing height minus the sitting height was calculated for each athlete. Finally, to obtain the estimated age at PHV, the chronological age was subtracted by the maturity offset score of each player. The maturity offset has been shown to be a valid and reliable measure to estimate the age at PHV [45]. However, it has been also shown that the maturity offset is more reliable when conducted within one year of PHV [45].

### 2D:4D ratio

The second- and fourth-digit length of both hands were measured [28]. Each player placed the right and left-hand palm on a scanner with the fingers kept 2 cm apart from each other. The image of player’s palms in the scanner was transferred to a computer and the Kinovea software was used to analyse fingers’ length. The second- and fourth-digit length were measured from the crease proximal to the palm to the tip of the digit.

The ratio of both fingers was calculated as the division of the second digit length by fourth length of both hands. The model of the scanner was the Scanjet (5590 HP Scanjet, USA) with an accuracy of 0.01 cm measurement of second and fourth finger to the tip of the finger. The difference of the Right 2D:4D by the Left 2D:4D (Right-Left 2D:4D) was calculated. The intra-observer reliability was assessed by the same observer two times a week apart. The intra-class correlation (ICC) for 2D:4D was 0.93 and 0.95, respectively.

### Statistical analysis

Tests of normal distribution and homogeneity (Kolmogorov–Smirnov and Levene’s, respectively) were conducted on all data before analysis. A paired sample *t*-test was used to determine differences as a repeated measures analysis in two conditions (Pre – Post) for physical fitness variables. Cohen d was used as the effect size indicator. To interpret the magnitude of the effect size, we adopted the following criteria [46]: d = 0.20, small; d = 0.50, medium; and d = 0.80, large. Posteriorly, the percentage change of physical fitness measures was calculated as follows: [100-(Pre*100)/Post].

The Pearson’s correlation coefficient r was used to examine the relationships between the maturity status estimation and physical fitness changes. The relationships between the 2D:4D and physical fitness changes were examined. Also, the relationships between training load measures of the whole season and physical fitness changes were considered in the analysis. To interpret the magnitude of these correlations, the following criteria was adopted [47]: r ≤ .1, trivial; 0.1 < r ≤ .3, small; 0.3 < r ≤ .5, moderate; 0.5 < r ≤ .7, large; 0.7 < r ≤ .9, very large; and r > .9, almost perfect. A regression analysis was used to examine which variable of maturity status and 2D:4D measures could be used to better explain the percentage of change of physical fitness measures with significant correlations. All data were analysed using the software Statistica (version 13.1; Statsoft, Inc., Tulsa, OK, USA) and the significance level was set at *p* < .05.

## Results

The descriptive statistics of maturity status estimation and 2D:4D measures are in Table 2.

**Table 2:** Maturity status and the 2D:4D at baseline

Maturity Status	Mean ± SD	Min.	Max.
Maturity-offset (years)	1.6 ± 0.5	0.28	2.84
Age at PHV (years)	13.4 ± 0.3	12.90	14.04
**2D:4D of both hands**	**Mean ± SD**		
Left 2D (cm)	7.6 ± 0.5	6.91	8.89
Left 4D (cm)	7.9 ± 0.5	7.11	9.05
LF2D:4D (cm)	0.9 ± 0.0	0.90	0.99
Right 2D (cm)	7.5 ± 0.5	6.95	8.96
Right 4D (cm)	7.9 ± 0.5	7.20	9.07
RF2D:4D (cm)	0.9 ± 0.0	0.90	1.01
R-L 2D:4D (cm)	0.0 ± 0.0	-0.04	0.05

*PHV* peak height velocity, *D* digit, *LF* left finger, *RF* right finger, *RL2D:4D* Right-Left 2D:4D

Descriptive statistics of physical fitness changes (pre-post assessments) are in Table 3.

**Table 3:** Physical fitness before and after the season (mean ± SD).

Physical Fitness	Pre	Min;Máx	Post	[Min;Máx]
Sit and Reach (cm)	37.8 ± 8.3	24.0;50.0	40.7 ± 9.0	25.0;56.0
Mod. 505 COD (sec)	2.1 ± 0.2	1.83;2.54	1.9 ± 0.1	1.67;2.23
Peak Power (AU)	708.0 ± 128.4	414.0;925.0	821.0 ± 106.4	568.0;947.0
VIFT (km/h)	16.7 ± 2.2	13.0;21.5	17.9 ± 2.6	13.0;22.0

**Fig. 2:**
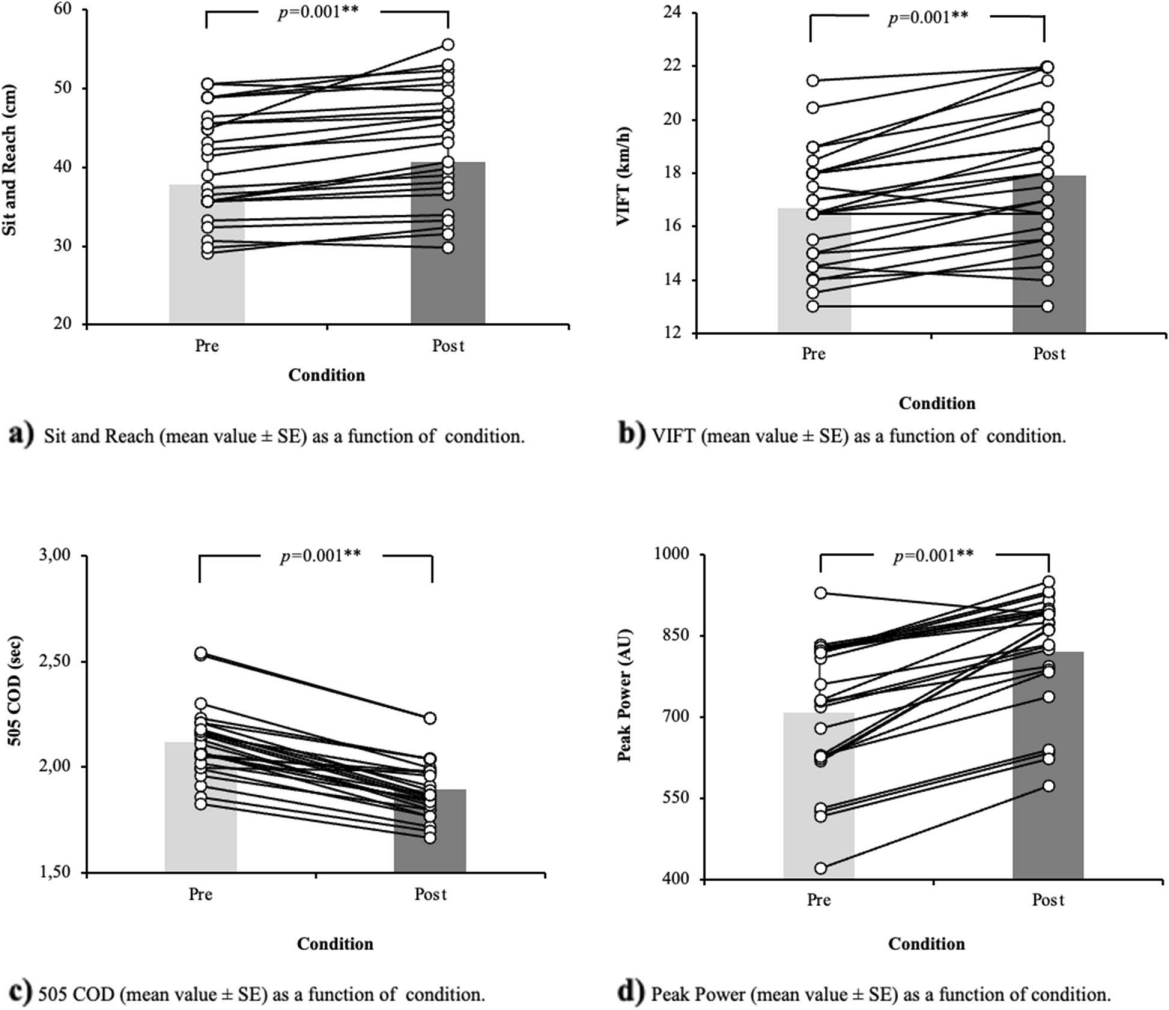
Between-period (pre-post) physical fitness differences

A paired measures *t*-test with participants’ physical fitness assessment revealed significant differences in Sit and Reach, 505 COD test, PP, and VIFT (*p* = .001, *d* = -0.32; *p* = .001, *d* = -0.47; *p* = .001, *d* = 1.40; *p* = .001, *d* = -0.95 and *p* = .001, *d* = -0.51, respectively). For more information, see Fig. 2.

**Fig. 3:**
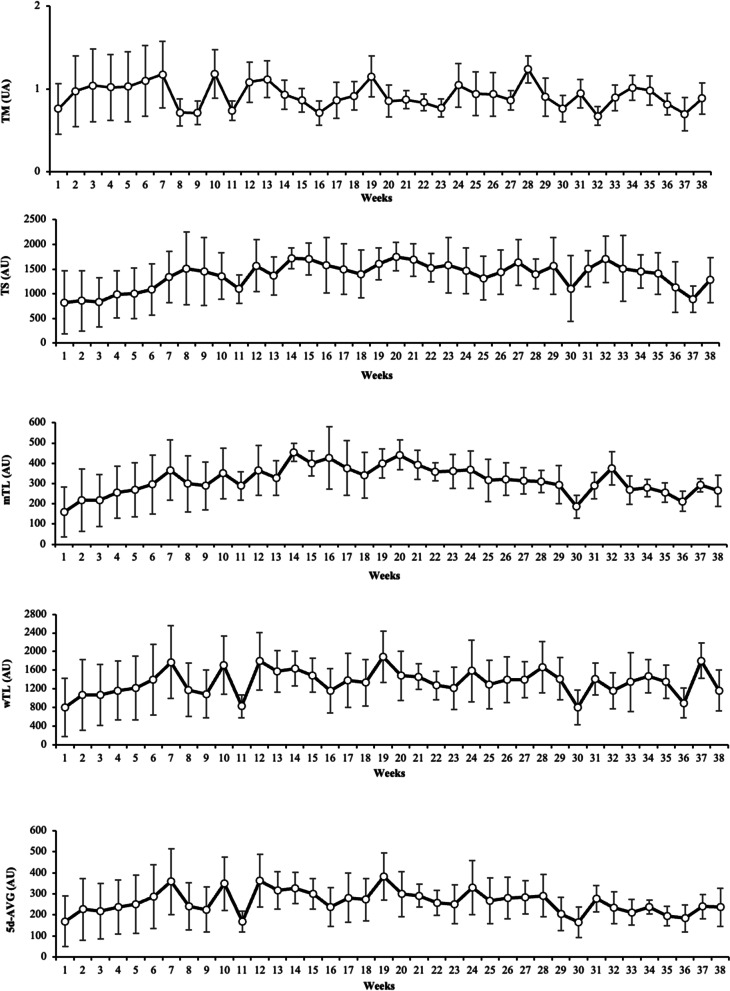
Weekly training load distribution across the season for TM: training monotony; TS: training strain; mTL: mean training load; wTL: weekly training load; and 5d-AVG: 5 day-average.

Figure 3 shows the weekly training load distribution during the season for all load variable information

A correlation analysis was performed between training load measures of the 38 weeks and the percentage of change of physical fitness measures (Table 4).

**Table 4:** Correlations between TL (entire season) and the changes of physical fitness

	S&R	VIFT	Mod.505 COD	PP
**TM**	*r* = .02 | *p* = .92	*r* = .15 | *p* = .47	*r* = .33 | *p* = .11	*r* = .17 | *p* = .43
**TS**	*r*=-.03 | *p* = .89	*r*=-.01 | *p* = .96	*r* = .09 | *p* = .68	*r* = .03 | *p* = .91
**mTL**	*r* = .15 | *p* = .48	*r* = .18 | *p* = .39	*r* = .20 | *p* = .34	*r* = .19 | *p* = .38
**wTL**	*r*=-.33 | *p* = .11	*r*=-.02 | *p* = .92	*r*=-.12 | *p* = .56	*r*=-.12 | *p* = .58
**5d-AVG**	*r* = .04 | *p* = .83	*r*=-.10 | *p* = .60	*r* = .36 | *p* = .07	*r* = .02 | *p* = .89

*TM* training monotony, *TS* training strain, *mTL* mean training load, *wTL* weekly training load, *5d-AVG* 5 day average, *S&R* sit and reach test, *VIFT*, final velocity of 30 − 15 intermittent fitness test, *Mod. 505 COD* modified 505 change of direction

The relationships between the 2D:4D ratio of both hands and physical fitness measures can be seen in Table 5.

**Table 5:** Correlations between 2D:4D, maturity status measures and physical fitness changes

2D:4D measures	S&R	VIFT	Mod.505 COD	PP
**Left 2D**	*r*=-.07 | *p* = .72	*r* = .01 | *p* = .96	*r*=-.39 | *p* = .05*	*r* = .04 | *p* = .82
**Left 4D**	*r*=-.11 | *p* = .57	*r* = .11 | *p* = .58	*r*=-.41 | *p* = .04*	*r* = .01 | *p* = .99
**LF2D:4D**	*r* = .10 | *p* = .60	*r*=-.24 | *p* = .23	*r* = .03 | *p* = .88	*r* = .10 | *p* = .61
**Right 2D**	*r*=-.02 | *p* = .92	*r* = .01 | *p* = .98	*r*=-.45 | *p* = .02*	*r* = .05 | *p* = .79
**Right 4D**	*r*=-.04 | *p* = .83	*r* = .05 | *p* = .79	*r*=-.44 | *p* = .03*	*r* = .01 | *p* = .94
**RF2D:4D**	*r* = .05| *p* = .78	*r*=-.09 | *p* = .66	*r*=-.06 | *p* = .74	*r* = .07 | *p* = .72
**R-L2D:4D**	*r*=-.04| *p* = .84	*r* = .15 | *p* = .46	*r*=-.13 | *p* = .52	*r*=-.01 | *p* = .95
**Maturity Status**	**S&R**	**VIFT**	**Mod.505 COD**	**PP**
**Maturity offset**	*r*=-.07 | *p* = .71	*r*=-.09 | *p* = .64	*r* = .41 | *p* = .04*	*r* = .22 | *p* = .28
**Age at PHV**	*r*=-.02 | *p* = .89	*r* = .10 | *p* = .61	*r* = .07 | *p* = .73	*r*=-.01 | *p* = .93

*PHV* peak height velocity, *D* digit, *RF2D:4D* right finger 2-digit:4-digit, *LF2D:4D*, left finger 2-digit:4-digit, *RL2D:4D* Right-Left 2D:4D, *TM* training monotony, *TS* training strain, *mTL* mean training load, *wTL* weekly training load, *5d-AVG* 5 day average, *S&R* sit and reach test, *30 − 15 IFT* 30 − 15 intermittent fitness test, *PP* peak power

* Denotes significance at *p* < .05

A multilinear regression analysis was performed to verify which variable of maturity status and 2D:4D measures could be used to better explain the percentage of change of physical fitness measures. It was found that maturity offset, Left 4D, Right 2D and Right 4D significantly predicted the Mod.505 COD (β = 0.41, *p* = .04; β = -0.41, *p* = .04; β = -0.45, *p* = .02; and β = -0.44, *p* = .03, respectively).

## Discussion

The aims of the present study were to analyse the physical fitness changes of youth football players after a full-season, and to examine whether physical fitness changes were explainable by maturity status estimation, 2D:4D and TL measures. The main findings were that from the maturity status measures (maturity offset and age at PHV) and from the putative biomarker for prenatal testosterone measures (2D:4D), only the maturity offset, and the second and fourth digits of both hands revealed moderate to associations with COD performance changes. On the other hand, neither maturity offset nor 2D:4D measures revealed significant relationships with the overall physical fitness changes. Also, from the reported significant correlations, the maturity offset, Left 4D, Right 2D and Right 4D measures significantly predicted COD performance changes.

There are several studies reporting physical fitness seasonal changes of youth football players [48, 49]. The present study revealed significant improvements of all the analysed physical fitness measures, from the beginning to the end of the season. Indeed, previous findings revealed that positive changes in physical fitness occurs after consistent football training programs in youth [48]. However, the consideration of maturity status as a variable that may influence such changes must be acknowledged [48]. A recent study [33] conducted on 23 under-16 football players, revealed that VO_2_max and Peak Power had significant changes between pre- and post-assessments, which is in concordance with the findings of the present study. Although some studies reported significant improvements in physical fitness after a football season, others revealed that these changes are not so straightforward as they may be dependent on baseline values [50, 51].

Our findings revealed that there were no correlations between all TL measures and physical fitness changes, which is congruent, at some extent, with previous studies conducted on adult football players [52]. For instance, a study conducted on 26 professional football players showed that TL obtained by the Banister’s training impulse method had a moderate correlation with VO_2_max changes (*r* = .46; [0.04; 0.74]) [53]. However, no significant relationships were reported for other physical measures such as strength-oriented measures [53]. On the other hand, our findings are in contrast with previous studies which reported that the accumulated TL had negative associations with changes in aerobic, vertical jump and sprint performance (*r* = -.51 to -0.64) [54, 55]. In contrast to the studies of Los Arcos et al. [54, 55], found that TL quantified by subjective measures were positively and largely correlated (*r* = .67–0.71) with aerobic performance changes [16]. These differences between studies may be attributed to the frequency and duration of training sessions, as well as different approaches of the training process.

Previous studies demonstrated the influence of biological maturation on physical fitness variations in soccer players [25, 56, 57]. For instance, it was previously reported in longitudinal studies that using the skeletal age as a marker of biological maturation can potentially explain aerobic performance and repeated sprint ability on youth football players [56, 57]. However, to measure skeletal age is needed high-cost equipment that is not accessible for the overall football youth academies. Given that, the use of other non-invasive methods such as the maturity offset is more common. However, our model revealed that both maturity offset and age at PHV explained small and inconsistent proportions of the observed physical fitness measures. Similar to our findings, a study conducted on 88 youth male football players revealed that using the maturity offset measure as a marker of biological maturation, explained small and inconsistent proportions of the observed physical fitness variations after a full-season [25]. Given that, other factors that were not included in our model may have influenced physical fitness and training load variations.

The 2D:4D method was previously reported to be a potential predictor of physical fitness variations [30]. Indeed, the 2D:4D ratio of the left and right hands of 24 under-17 football players, had negative large correlations with VO_2_max variations (*r* = -.55, *p* = .005; *r* = -.50, *p* = .013) [30]. In the present study, no correlation was found between 2D:4D measures and the velocity reached at the final stage of the 30-15IFT. Although the 30-15IFT is categorized as an aerobic test, in reality, the test measure maximal cardiorespiratory function, anaerobic capacity, neuromuscular function and the ability to recover during intermittent exercise [58].

Interestingly, in the present study, the 2D and 4D of the left and right hands (i.e., without considering the ratio) had negative moderate correlations with COD variations. On the other hand, when considering the 2D:4D ratio of both hands, none of them presented significant correlations with physical fitness measures. This is in contrast with other studies that showed that players with lower 2D:4D ratio of both hands had significantly greater aerobic and anaerobic performance [59, 60]. However, the above-mentioned studies analyzed the VO_2_max and jump performance. Although we also measured maximal cardiorespiratory function, anaerobic capacity, neuromuscular function, the tests we used were different from other research [59, 60]. This fact may have contributed for this contrasting evidence. Still, from our findings, it seems that analyzing the finger lengths without calculating its ratio show that players with lower 2D and 4D lengths are better at performing COD tasks.

The present study had some limitations. The main limitation refers to the small sample size used. The fact that only one male team was included in the sample is another main limitation. However, in professional youth football competitions, the use of more than one team is a major concern for both coaches and practitioners. Another limitation to consider is the fact that we conducted indirect finger length measurements. According to a previous study, indirect measures of finger length through the use of scanners may cause small distortions of finger length that are not equal for the second digit and fourth digit [61]. It must be considered the fact that the 2D:4D ratio may not correctly normalize for 4D length uniformly [32]. However, the use of a simple non-invasive and cost-free measure as the 2D:4D ratio to predict physical fitness changes, surpasses the above-mentioned limitations when coaches and clubs do not have access to gold standard equipment. Finally, for the quantification of TL measures, we used the question “how intense was your session?” instead of using the original from Borg’s CR-10 scale [34]. However, this choice was made on the basis of the linguistic barriers of the sample used. Still, relevant studies also used the question used in the present study [25, 27]. Future studies should use larger sample sizes and examine the associations between different predictive maturity status and objective internal training load variations, such as heart rate-based measures.

## Conclusion

The overall physical fitness measures revealed significant changes from the beginning to the end of the season. The maturity status estimations and the 2D:4D measures seem not to be useful to predict overall physical fitness changes after a youth football season. However, the maturity offset and the second and fourth digits of both hands may constitute a relevant role to predict COD performance changes across a youth football season.

## Data Availability

The datasets generated and analysed during the current study are not publicly available due to ethical restrictions, however are available from the corresponding author on reasonable request.

## Abbreviations

COD: Change-of-direction
VIFT: Final velocity reached in the last stage of the 30-15 intermittent fitness test
s-RPE: Session-rate of perceived exertion
TM: Training monotony
TS: Training strain
wTL: Weekly training load
mTL: Mean training load
5d-AVG: 5-day average
PP: Peak power
FI: Fatigue index
PHV: Peak height velocity
D: Digit
RF2D:4D: Right finger 2-digit:4-digit
LF2D:4D: Left finger 2-digit:4-digit
